# ZmBAK1 confers maize resistance to *Gibberella* stalk rot caused by *Fusarium graminearum* via activating PAMP-triggered immunity

**DOI:** 10.1080/15592324.2025.2502739

**Published:** 2025-05-12

**Authors:** Fugui Xie, Yali Sun, Huilan Zhang, Junjie Cui, Qing Wang, Xiquan Gao

**Affiliations:** a State Key Laboratory of Crop Genetics & Germplasm Enhancement and Utilization, Nanjing Agricultural University, Nanjing, P. R. China; b College of Agriculture, Nanjing Agricultural University, Nanjing, P. R. China; c Jiangsu Collaborative Innovation Center for Modern Crop Production, Nanjing Agricultural University, Nanjing, P. R. China; d College of Life Sciences, Henan Normal University, Xinxiang, Henan, P. R. China

**Keywords:** *Gibberella* stalk rot, maize, ZmBAK1, PAMP-triggered immunity, cell death

## Abstract

Gibberella stalk rot (GSR) caused by *Fusarium graminearum* is one of the most devastating diseases of maize, not only seriously affecting its yield and the application of mechanized harvest technology but also producing a variety of toxins, thus seriously impacting the food safety. BAK1 (Brassinosteroid-Insensitive 1-Associated Receptor Kinase 1, BAK1) is the well-studied co-receptor of PRRs (Pattern Recognition Receptors), which is involved in the regulation of growth and development regulation as well as the response to diverse biological stresses. However, little is known about the role of BAK1 in the interaction between maize and pathogens, especially in maize against GSR. In this study, we found that ZmBAK1 (*Zm00001d037297*) was located at the cytoplasmic membrane. Furthermore, *ZmBAK1* was induced by multiple PAMPs (Pathogen-Associated Molecular Patterns), while PTI (PAMP-Triggered Immunity) response including ROS (Reactive Oxygen Species) burst and callose deposition, as well as cell death, and immune gene expression was weakened in *bak1* mutant upon PAMP treatment. On the contrary, the ROS production and cell death in *BAK1*-OE were significantly stronger than wild type. Furthermore, *bak1* mutant is more susceptible to GSR, while *BAK1*-OE is more resistant, compared to wild types. Taken together, our data suggested that ZmBAK1 plays a positive role in maize GSR resistance, likely via activating PTI signaling pathway.

## Introduction

Maize (*Zea mays* L.) is one of the major crops worldwide, whereas it often encounters the attack by different types of diseases. Among the various devastating diseases, maize stalk rot, an important type of soil-borne diseases caused by *Fusarium* spp., usually causes significant yield reduction or even complete failure.^1,2^ The yield loss of maize due to stalk rot is normally 10%−20% in general years, even over 80% in serious cases. Particularly, maize stalk rot caused by *F. graminearum*, also known as Gibberella stalk rot (GSR), not only impacts the production and quality of maize but also retarders the application of mechanical harvesting technology in maize, thus affecting greatly the food safety of maize.^3^

It has been widely known that the resistance to maize GSR is a minor-effect trait controlled by QTLs (Quantitative Trait Loci). At present, several QTLs for GSR resistance have been reported; however, only four QTLs (*qRfg1*, *qRfg2*, *qRfg3* and *qRgsr8.1*) have been systemically characterized.^4–6^ Furthermore, four candidate genes, including *ZmAuxRp1* ^6^ encoding an auxin responsive protein, *ZmCCT*,^5^ *Zm953* and *Zm972* encoding an auxin responsive factor and a resistance protein RPP13 in the *Rgsr8.1* region,^7^ respectively, were successfully cloned. Moreover, through integrative omics and reverse genetics approaches, several potential resistance genes, including *ZmPrX5*,^8^ *ZmWRKY83*,^9^ *ZmHIR3*,^10^ as well as jasmonates and oxylipin pathways,^8,11^ have been reported to play important roles in GSR resistance. However, the resistance to maize stalk rot is inherited and controlled by multiple genes with additive effects, and the mechanisms underlying GSR resistance are complicated, which has not yet been fully investigated.

To prevent the invasion of pathogens, plants have evolved two different types of innate immunity, e.g., pathogen-associated molecular pattern (PAMP)-triggered immunity (PTI) and effector-triggered immunity (ETI).^12^ Most pathogens are able to secrete a series of protein called effectors, which can promote and facilitate their pathogenesis. Numerous effectors have been shown to play important roles in suppressing PTI.^13^ ETI is characterized by the direct or indirect recognition of effectors by Nucleotide binding leucine-rich repeat receptors (NB-LRR, NLRs) in plant cells. Furthermore, ETI was also reported to overlap with PTI to simultaneously activate immunity.^14^ In maize, two NLR genes *RppC* and *RppK* have been successfully cloned for major QTLs of resistance to maize gray leaf spot. RppC and RppK could recognize the corresponding effectors AvrRppC and AvrRppK, respectively, to trigger strong ETI response.^15–17^ Moreover, R protein Rp1 has been extensively characterized for its role in activation of ETI in maize.^18^ In addition, maize NLR gene *Rcg1* confers the resistance of anthracnose stalk rot.^19^

It has also been known that PRRs often require co-receptors, such as BAK1, one member of somatic embryogenesis receptor kinases (SERKs), to perceive and transduce the signals from PAMPs to downstream immune response. Most SERKs encodes leucine-rich repeat (LRR) receptor-like kinases (RLKs) with an extracellular LRR domain, a transmembrane domain, and an intracellular kinase domain.^20^ BAK1 has been reported to form receptor complexes with BRI1 (BRassinosteroid-Insensitive 1) to regulate diverse physiological processes associated with brassinosteroid signaling, including vascular bundle differentiation, stem extension, flowering, flower organ abscission, fertility, and senescence.^21–23^ Furthermore, BAK1 has also been widely reported to play essential roles in plant immune responses,^24,25^ mostly via interacting with and phosphorylating the receptor kinase flagellin-sensitive 2 (FLS2), as well as the cytoplasmic receptor kinase botrytis-induced kinase 1 (BIK1).^26–28^ In addition to FLS2, BAK1 also forms complexes with other receptor kinases, including PEP1 RECEPTOR 1 (PEPR1/2), EF-TU RECEPTOR (EFR) and BAK1-INTERACTING RECEPTOR-LIKE KINASE 1/2 (BIR1/2), as well as BAK1-Like 1 (BKK1), to activate PRR immune signaling pathways and HR-like cell death.^29–31^ BAK1 was also reported to interact with BON1 and CNGC20 to modulate cell death.^30,32^ Furthermore, BAK1 and BKK1 were also reported to serve as guardees for NLRs-mediated autoimmune response, typically characterized by spontaneous cell death caused by the disruption of BAK1 and BKK1.^33^ Similarly, BAK1 and BIR3 perturbation are guarded by the TIR-NLR protein CONSTITUTIVE SHADE AVOIDANCE 1 (CSA1),^34^ suggesting the multifaceted and complex functions of BAK1 in both PTI and ETI signaling pathways. Moreover, using a co-expression network analysis approach, a maize BAK1 gene (GRMZM2G145440) was found to be highly co-expressed with the phytoalexin biosynthesis genes and a fungal-induced RLK gene *Fi-RLPK* in response to fungal pathogens *C. heterostrophus* and *F. graminearum*.^35^ These data together suggest the potential roles of BAK1 in regulating both growth and development, as well as defense against pathogens in maize.

Despite the above findings reported, very little is known about the role of BAK1 in maize interaction with pathogens, especially in resistance to *F. graminearum*. This study analyzed the role of a maize *ZmBAK1* gene in maize resistance to GSR. Using UniformMu mutant and overexpressor lines, GSR phenotypes were functionally investigated. The results together showed that ZmBAK1 plays a positive role in regulating GSR immunity, which was likely exerted through activation of PTI in maize, suggesting a possibly conserved ZmBAK1-mediated innate immunity signaling pathway in maize, which might provide potential gene resources for maize disease resistance breeding and novel insights into understanding the molecular mechanisms of maize innate immunity to fungal pathogens.

## Materials and methods

### Plant materials and culture

Wild type line W22 and transposon insertion mutant line *zmbak1* in W22 genetic background (UFMu -02,199; ID: *Zm00001d037297*) were provided by Maize Genetics Stock Center (Figure S2a).

Overexpressor line of *ZmBAK1* was generated in KN5585 background (Figure S2b). The transformation service was provided by Weimi Biotechnology Co., Ltd (Changzhou, China).

GSR resistant line K09 and susceptible line A08 that have been characterized for the contrast GSR phenotypes in a previous work.^10^

The seeds of above maize lines were sown in the substrate soil (Xingnong Fertilizer Company, Jiangsu Province, China) in long pots made with PVC cylinders (5 cm in diameter and 20 cm in depth), with 5 seeds per cylinder. The seedlings were grown on a light shelf, with a light cycle of 14 h of light/10 h of darkness, at a temperature of 26 ± 2°C, and cultured for about 12–14 d until use.

The seeds of *N. benthamiana* were sown in the substrate soil (Xingnong Fertilizer Company, Jiangsu Province, China) in garden pots. The seedlings at the stage when two fully true leaves emerged were transplanted into new pots containing same soil as above, and grown in a growth chamber under the condition with 16 h of light, 8 h of darkness, and a temperature at 22°C for 1 month until use.

### Fungal strains, inoculum preparation, artificial infection and disease phenotype evaluation

*Fusarium graminearum* strain 0609, kindly provided by Prof. Mingliang Xu at China Agricultural University, was cultured and maintained on PDA media as regular. The fungal inoculum was prepared according to the described procedure in a previous work.^10^

For GSR seedling assay, the seeds of various maize lines were sown in the soil and cultured for about 12–14 d as described above. Then, a 20 μL of *F. graminearum* spore suspension were gently applied onto a wounding site made by a pin on the seedling stem, and the tray was subsequently covered with the PressInSeal Saran wrap (GLAD, Canada) to maintain the humidity.^10^ Fungal inoculation, phenotypic evaluation and disease grade evaluation of GSR on adult plants in the field were conducted as previously described.^11^ Briefly, at the full flowering stage of maize, a hole was made using a drill in the third node of stem above the ground at an inclined direction of 45 degrees, reaching a depth at half of the stem. Subsequently, a 200 μL of fungal spore suspension at the concentration of 1 × 10^6^/ml were injected into the wound with a veterinary inoculator. The wounding site was subsequently sealed using solid paraffin wax. The disease symptoms were scored at 14 d post inoculation (dpi), using a 7-level scale ranging from 1 (most resistant) to 7 (most susceptible).

### Quantitative real-time PCR (RT-qPCR) analysis

RNA samples from all maize stalk tissues at 0, 6, 12, and 24 h post-inoculation, respectively, were isolated and converted into cDNA using a reverse transcription kit (Yeasen, Shanghai, China, CAT: 11141ES60). SYBR® Premix Ex Taq ™ II (TaKaRa, Dalian, China, RR820A) was used for qRT-PCR analysis. The relative gene expression of *ZmBAK1*, *ZmPR1*, *ZmPAL3*, *ZmMYC7* to the reference gene was calculated using 2^−ΔΔCt^ method.^36^ The transcript level of *ZmActin* (*Zm00001d01227*) was used for internal normalization. Three independent replicates were performed. RT-qPCR primers are listed in Supplementary Table S1.

### Subcellular localization

The subcellular localization of ZmBAK1 was performed by the transient transformation in *Nicotiana benthamiana* according to a previous work with slight modification.^37^ In brief, the candidate gene fragment was ligated into pCambia1305.1-GFP vector, and the corresponding plasmid with correct sequencing was transformed into *Agrobacterium tumefaciens* (GV3101). The *Agrobacterium* clone was selected based on sequencing result and added to 500 µL of LB liquid medium containing Kanamycin+Rifmycin (50 mg/ml), incubated at 28℃, and centrifuged at 200 rpm for 8 h. One hundred microliters of bacterial solution added to 20 ml of LB^k+rif^ liquid medium, shaken at 28℃ and centrifuged at 200 rpm, until OD_600_ reaching 0.8–1.0. The solution was then centrifuged at 4000 rpm for 10 min and the supernatant was discard. The solution was then adjusted to OD_600_ = 1.0 by adding injection buffer, and kept at room temperature for 2 h. The bacterial solution was injected on the backside of leaves of *N. benthamiana* plants at third to fifth true leaves stage (approximately 2–3 weeks after the emergence of first true leaf). The infiltrated leaves were then wrapped with Saran-wrap, cultured in the dark for 36 h. The subcellular localization of BAK1 protein was examined under a laser confocal microscope.

### Treatments of PAMPs

To apply PAMPs exogenously, flg22 and chitin powder was dissolved and diluted using ddH_2_O into 100 μM and 500 μg/ml, respectively. The maize seedlings at 8 d post sowing (at the emergence of third true leaf) were sprayed with freshly prepared PAMPs solution until the water drops form on leaf surface. Control plants received same amount of ddH_2_O. The plants were kept at 24℃ with approximately 70% of humidity for 48 h, and leaf samples were harvested at different designated time points.

### *Detection of cell death in maize stalk infected with* F. graminearum

To visualize the cell death phenotype upon fungal infection, approximately 2 cm length of segment was excised from the seedling stem at the inoculation site. The cotyledons and first true leaf of maize seedling sprayed with PAMPs were detached. The tissues harvested were immediately immersed in 10 ml of trypan blue staining solution containing 0.3 mg/ml trypan blue dissolved in lactophenol (lactic acid: glycerol: liquid phenol: distilled water 1:1:1), stained, decolorized, and fixed for microscopic observation according the procedure described previously.^10^

### ROS burst determination

ROS burst was quantified using a multi-plate reader (GloMax Discover, GM3000, Promega) as described before.^10^ In brief, two fully expanded leaves of both *zmbak1* mutant and *ZmBAK1*-OE, as well as their corresponding WT plants were collected at V2 stage, and leaf discs were excised at the same position of each leaf with a punch. To eliminate the effect of wounding, leaf discs were subsequently placed in a 96 well plate containing 200 μL of ddH_2_O per well, incubated at 23℃ for 14 h under weak light by gentle shaking. The ddH_2_O was sucked out, and 100 μL of detection solution (containing 35.4 μg/mL luminol, 10 μg/mL horseradish peroxidase, 0.1 μM chitin) was immediately added in each well, upon immediate reading in the multiplate reader with a reading time interval at 1 s for each well. Three leaf discs were used for one treatment and at least three replicates were performed.

### Determination of callose deposit

The leaf upon PAMPs treatment was harvested and immediately transferred into a 50 ml centrifuge tube containing 45 ml decolorization solution (anhydrous ethanol: acetic acid = 3:1), and vacuumed for 30 min, then incubated at room temperature for 12 h at 70 rpm in a shaker. The solution was replaced for 2 ~ 3 times until the leaf tissue is completely transparent. After the tissue was completely decolorized, the decolorization solution was then replaced with 150 mm K_2_HPO_4_ buffer for rehydration for 30 min, following twice washing with fresh K_2_HPO_4_ buffer. Finally, the rehydration buffer was replaced with 0.3% aniline blue staining solution (0.3% aniline blue (Sigma) powder was dissolved in 150 mm K_2_HPO_4_ buffer, pH = 9.3), and staining for overnight at room temperature. The staining solution was finally replaced for washing twice with and kept in fresh K_2_HPO_4_ buffer.^25^ Callose was examined using a fluorescence microscope under the excitation of ultraviolet light. A minimum of four biological replicates per treatment were performed, with each replicate consisting of at least 10 technical repeats. Furthermore, at least 10 microscopic fields per leaf sample was examined under microscope.

### Detection of H_2_O_2_ accumulation

To determine the H_2_O_2_ accumulation, the first true leaf and cotyledons of approximately 2-week-old maize seedling were excised and quickly immersed in DAB staining solution (1 mg/ml, pH 3.8), then subjected to vacuum filtration for 30 min, followed by incubating at room temperature in the dark for 8 h. Upon staining, the tissues were heat boiled in 95% ethanol for about 15 min for decolorization. The decolorized tissues were transferred into 20% of glycerol.^38^ The staining was visualized and recorded under a microscope. Three biological replicates per treatment were performed per treatment, with at least 10 seedlings each biological replicate ensured.

#### Statistical analyses

All statistical analyses were performed using GraphPad Prism 8.0 software. Reactive oxygen species (ROS) burst data and callose deposition data were processed using Student’s t-test. The data for GSR phenotype upon infection with *F. graminearum* were processed using Tukey’s ANOVA.

## Results

### ZmBAK1 subcellular localization

**Figure 1.**
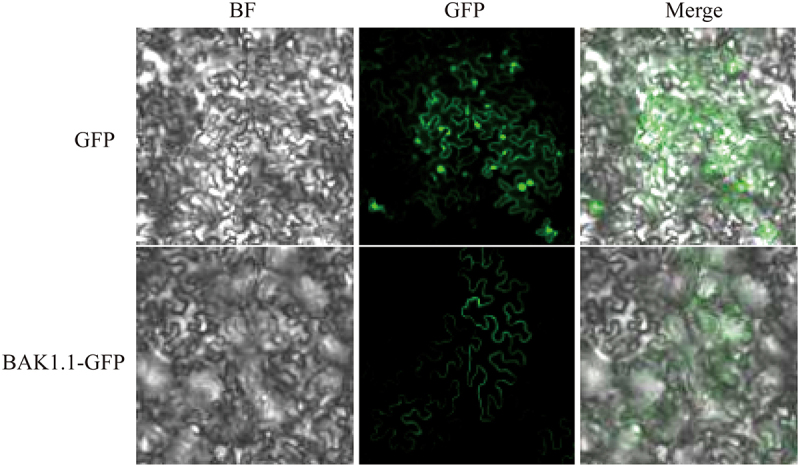
Subcellular localization of ZmBAK1. BF stands for bright field, GFP stands for green fluorescent field of vision, the merge indicates the overlapping field of green fluorescence and bright. The experiment was conducted by three biological replicates, with consistent results obtained.

To explore the subcellular localization of *ZmBAK1*, ZmBAK1-GFP was constructed by inserting ZmBAK1 into pCAM1305.1-GFP vector and constructed with GFP tag. The construct was then mixed with *Agrobacterium* and infiltrated into the leaves of *Nicothiana benthamiana*, and the GFP signal was observed under a laser confocal microscope at 48 h post transient expression. While the GFP signal of empty vector was detected in the nucleus and cell membrane of the *N. benthamiana* epidermis, ZmBAK1-GFP protein was only expressed in the cell membrane (Figure 1), suggesting that ZmBAK1 is located at the cell membrane.

### Involvement of ZmBAK1 in activation of PTI signaling pathway

**Figure 2.**
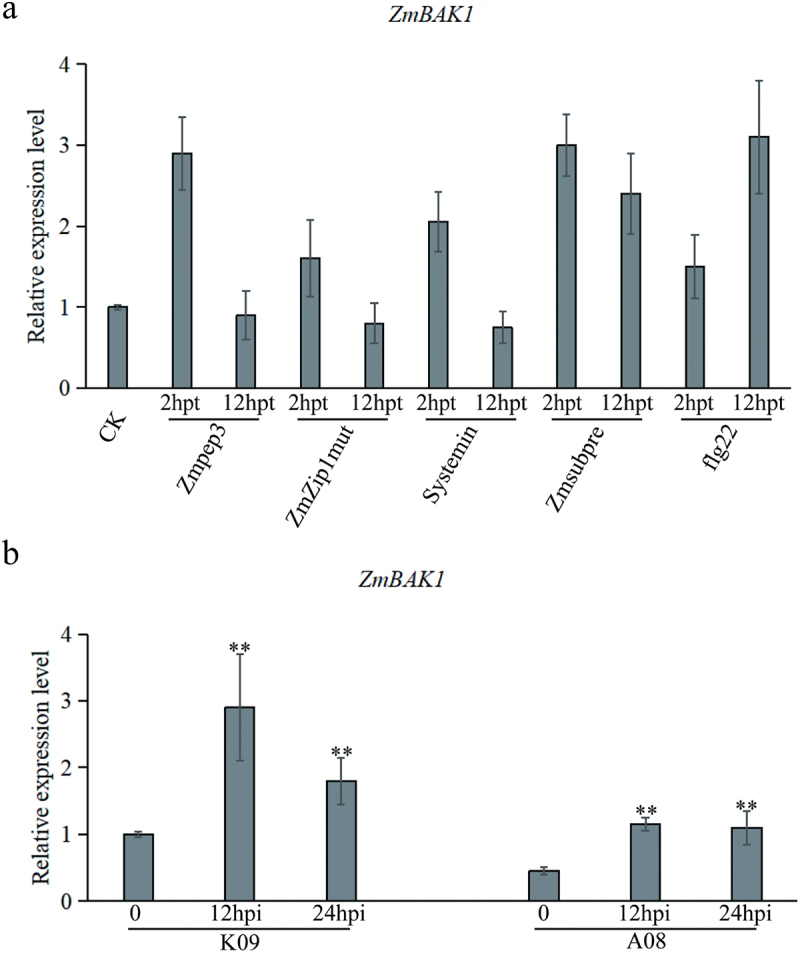
Expression of *ZmBAK1* under different treatment conditions (a) expression of *ZmBAK1* after treated with different endogenous peptides and PAMPs in maize B73 protoplasts at 2hpt and12hpt. (b) expression of *ZmBAK1* at different time points after inoculation with *F. graminearum* in resistant K09 and susceptible A08 lines. Among (a), CK represents water treatment, ZmPep3, ZmZip1mut and ZmSubpre are different endogenous small peptides from maize, respectively; systemin represents systemins, flg22 represents flagellin from bacteria. Among (b), K09 represents the stalk rot resistant material, A08 represents the stalk rot susceptible material, and 12 hpi, 24 hpi represent the inoculation time of *F. graminearum*, respectively. Three biological replicates were performed per treatment, with consistent results obtained. *indicates a significant difference at the 0.05 level (t-test), and **indicates a significant difference at the 0.01 level.

To understand whether and how ZmBAK1 is involved in maize immune response, the expression levels of *ZmBAK1* were determined using RT-qPCR in the protoplasts of inbred line B73 upon treatment with different PAMPs and peptides, including Zmpep3, ZmZip1 mut, Systemin, Zmsubpre and flg22. The treatments with all PAMPs and peptides induced the expression of *ZmBAK1* in maize protoplasts at 2 h post treatment (hpt), then declined at 12 hpt, except that for flg22 at 12 hpt, which induced higher expression of *ZmBAK1* compared to that at 2 hpt (Figure 2a). To reveal whether *ZmBAK1* is involved in the immunity to GSR, a pair of previously identified maize inbred lines with contrast GSR phenotype were infected with *F. graminearum*, and the expression of *ZmBAK1* was measured at different time points post infection. The results showed that although *ZmBAK1* expression was up-regulated in resistant line K09, and the susceptible line A08, at both 12 and 24 hpi, the expression in resistant line was higher than that in susceptible line (Figure 2b).

**Figure 3.**
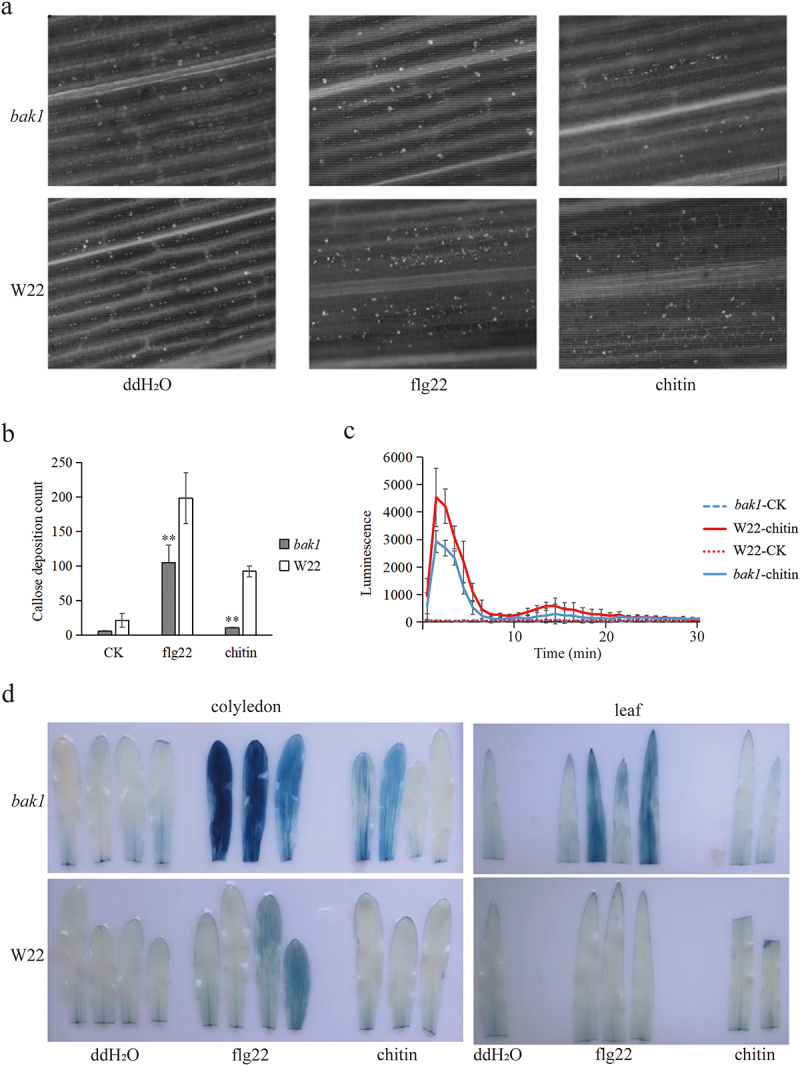
ZmBAK1 participates in PTI immune signaling pathway. (a) callose deposition of maize leaves of W22 and *zmbak1*. (b) amounts of callose deposition in maize leaves of W22 and *zmbak1*. (c) chemiluminescence assays to show ROS burst induced by chitin in the wild-type and *zmbak1* leaves. (d) cell death of maize seedling leaf after exogenous sprayed with flg22 and chitin at 48 hpi. The concentration of chitin was 500 μg/ml. The blue spots on a leaf were defined as cell death. All treatments of the two materials had three biological replicates, and each biological replicate ensured at least 10 seedlings. *indicates a significant difference at the 0.05 level (t-test), and **indicates a significant difference at the 0.01 level.

To further understand the role of ZmBAK1 in maize innate immunity, the ROS burst, cell death and callose deposition in the mutant and overexpressor plants of *ZmBAK1* and their corresponding wild type lines W22 and KN5585 were examined upon treatment with PAMPs, flg22 and chitin, respectively. The results showed that while both flg22 and chitin could enhance callose deposition in both WT and *zmbak1* mutant, the callose production was much lower in the mutant compared to WT (Figure 3a–b). In addition, the ROS burst of wild type W22 was significantly higher than that of *zmbak1* mutant after exogenous treatment with chitin (Figure 3c). On the contrary, ROS burst in *BAK1-*OE was significantly higher than that in wild type (Figure S1). Furthermore, it was found that strong cell death was detected in both the cotyledon and true leaf of *zmbak1* mutant plant upon treatment with flg22 for 48 h, whereas no obvious cell death was found in leaf tissue of wild type W22 plants. Chitin treatment only resulted in slight cell death in the cotyledons, but not in the true leaves of the mutant (Figure 3d). However, it should be noted that the cell death in cotyledons is relatively stronger than that in true leaf. Above results together suggested that BAK1 could activate the PTI signaling pathway in maize.

### ZmBAK1 positively regulates GSR resistance

**Figure 4.**
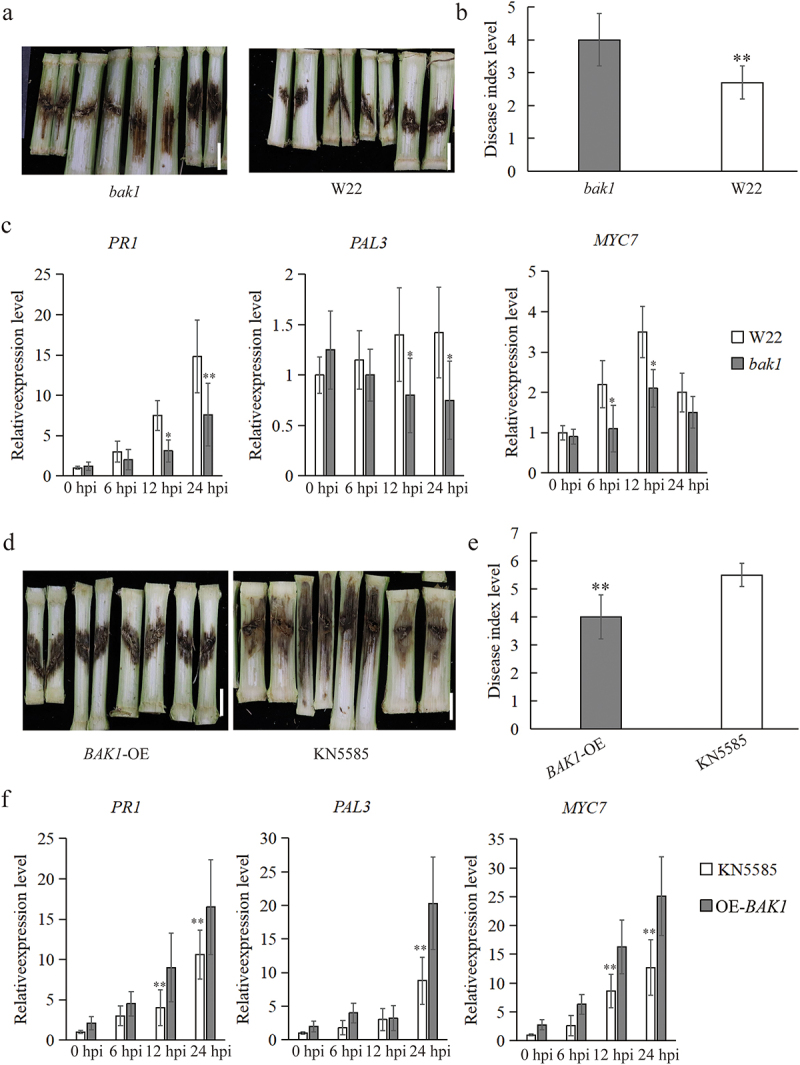
ZmBAK1 positively regulates GSR resistance. (a) GSR phenotypes of *zmbak1.19* vs wild type W22. (b) quantification of GSR disease index level of *zmbak1.19* and W22 shown in (a). (c) quantification of expression levels of selected immune genes in W22 and *zmbak1.19* at 6hpi, 12 hpi and 24 hpi upon infection with *F. graminearum*. (d) GSR phenotypes of *ZmBAK1.1*-OE vs wild type KN5585. (e) quantification of GSR disease index level of *ZmBAK1.1*-OE and KN5585 shown in (d). (f) quantification of expression levels of selected immune genes in *ZmBAK1.1*-OE and KN5585 at 6hpi, 12 hpi and 24 hpi upon infection with *F. graminearum*. three biological replicates were performed per treatment, with consistent results obtained. *indicates a significant difference at the 0.05 level (t-test), and **indicates a significant difference at the 0.01 level.

In order to understand whether ZmBAK1 plays a role in GSR resistance, the Mu-insertional mutant and overexpressor lines of *ZmBAK1* was infected with *F. graminearum* for phenotypic identification of GSR in the field. The results showed that *zmbak1* mutant plants exhibited significantly higher susceptibility to GSR, compared to that of wild type W22 plants (Figure 4a–b). The gene expression profiling showed that although the expression levels of *PR1*, one of the representative marker genes of SA (salicylic acid) signaling pathway, and *MYC7*, the representative marker gene of JA (jasmonic acid) signaling pathway, were all induced in both WT and mutant plants, in response to *F. graminearum* infection, their expression levels in mutant were significantly lower than that in wild type W22. Another SA signaling pathway marker gene *PAL1* was slightly induced in WT, although not significant, but significantly higher compared to that in the mutant (Figure 4c). On the contrary, compared with WT KN5585, *BAK1*-OE plants displayed significantly higher levels of GSR resistance (Figure 4d–e). In line with this result, *BAK1*-OE showed significantly higher expression levels of *PR1*, *PAL1* and *MYC7* (Figure 4F). These data together revealed that ZmBAK1 plays a positive role in GSR resistance.

## Discussion

Plants sense environmental changes through receptors on the cell membrane and transduce corresponding signals to regulate diverse physiological and biochemical processes. PTI triggered by the perception of PAMPs by PRRs and their accompanying co-receptors confer the basic immunity of plants to a wide range of pathogens. PTI is often typical of multiple hallmarks, including calcium ion influx, ROS burst, and callose deposition, as well as activation of immune genes.^39–41^ In addition, PAMPs also induce significant PCD,^42,43^ indicating that PCD is likely also an indicator of PTI in responsive to fungal elicitor and pathogen invasion.

BAK1 is a multifunctional RLK, which has been widely known to function in regulating plant growth and development, cell death, and immune signaling pathways.^25,44–46^ One of the most important roles of BAK1 is involved in activation of PTI immunity signaling pathway as a co-receptor of PRRs. There are very limited reports about maize PTI signaling pathways. Using a maize recombinant inbred line (RIL) population, a major QTL associated with flg22- and chitooctaose-triggered ROS production was identified on chromosome 2.^47^ A novel maize RLCK, BIK1-LIKE KINASE 1 (ZmBLK1) confers the resistance to Goss’s wilt caused by *Clavibacter michiganensis* subsp. *nebraskensis*.^48^ BIK1 was known to be an essential component in PTI signaling pathway, through associating with and phosphorylating FLS2 and BAK1, and also being phosphorylated by BAK1, thus transducing the PRR signals to downstream component to activate PTI.^49^ Thus, it is reasonable to presume that BAK1 may play an essential role in BIK1-mediated immunity in maize.

To affirm whether BAK1 is involved in maize innate immunity, PTI hall markers were examined in using *zmbak1* mutant and *OE-BAK1* lines upon treatment with flg22 and chitin. The results showed that ROS burst was significantly inhibited in *zmbak1* mutant after chitin treatment (Figure 3c). On the contrary, ROS burst was significantly increased in OE*-BAK1* (Figure S1). Moreover, callose deposition was suppressed in *zmbak1* compared to that of the control (Figure 3a–b). Interestingly, a difference of cell death between cotyledons and true leaf was observed. One of the possible reasons could be due to the organ-specific spatial and temporal pattern of cell death occurred in different organs, as it has been known that the cotyledons are formed upon embryogenesis, whereas true leaves are generated upon the cell division of shoot apical meristem.^50^ This hypothesis is also supported by a previous finding in maize that earlier PCD was observed on scutellum and coleoptile, yet not detected in leaf primordia.^51^ These results indicated that the PTI immune response triggered by chitin in maize presumably depends on ZmBAK1. This is contrary to the downstream signal transduction mediated by CERK1 sensing chitin in Arabidopsis that does not depend on SERK3/BAK1.^29,52,53^ In addition, the GSR resistance of *zmbak1* mutant was significantly suppressed (Figure 4a–b), whereas that of *BAK1-*OE was significantly improved (Figure 4d–e), suggesting that ZmBAK1 plays a positive role in maize GSR resistance. In supporting this finding, a most recent work showed that maize BAK1 could function as the co-receptor of a membrane-localized G-type lectin receptor kinase ZmLecRK1 to confer the maize resistance to stalk rot caused by *Pythium aphanidermatum*, further ratifying the importance of maize BAK1 in the resistance against diverse pathogens.^54^

At present, there are a number of reports about that BAK1 is involved in the process of crop disease resistance, such as rice blast, bacterial wilt, cotton Verticillium wilt, etc.^24,55,56^ The genes related to the hormone signaling pathway were significantly downregulated after inoculation in *zmbak1* mutant (Figure 4c). On the contrary, the expression of immune genes in *BAK1-*OE line was increased significantly after inoculation (Figure 4f). Furthermore, considering that *ZmBAK1* was also induced by a variety of elicitors (Figure 2a), it is likely that ZmBAK1 plays an important role in the activation of PTI response, probably through the downstream SA- and JA-related immune signaling pathways in GSR resistance.

BAK1 also plays an essential role in modulating plant cell death.^46^ While the cell death in maize seedling leaves was induced by exogenous flg22 and chitin treatment, much stronger cell death was detected in *zmbak1* mutant upon PAMP treatment (Figure 3d). Previous studies have shown that *F. graminearum* is a hemibiotroph pathogen, which is dominated by biotrophic lifestyle at early stage and necrotrophic lifestyle at late stage.^57^ The biotrophic pathogens prefer to live in the host living tissues, whereas necrotrophic pathogens prefer to live in dead tissues.^58^ These results suggest that ZmBAK1 may negatively regulate cell death triggered by PAMPs, suggesting that the immune response activated by ZmBAK1 is distinct in response to different bacterial and fungal PAMPs.

## Conclusion

**Figure 5.**
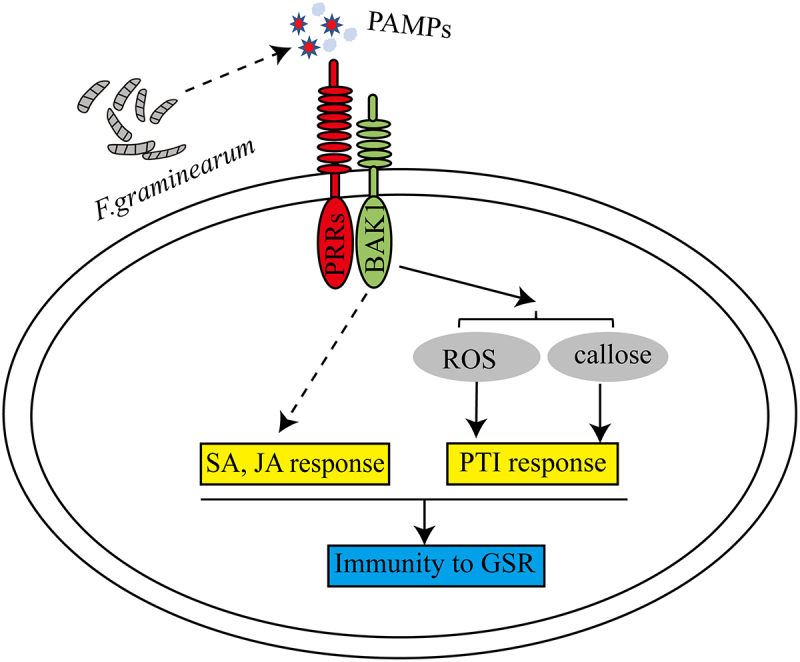
Working model for ZmBAK1-mediated immunity to GSR in maize. When maize is infected with *F. graminearum*, the PAMPs released by *F. graminearum* can be recognized by the uncharacterized PRRs in maize, while ZmBAK1 functions as a co-receptor of PRRs, triggering downstream immune signaling. Upon the perception of external stimuli, ZmBAK1 triggers PTI immune responses, including ROS accumulation and callose deposition. Meanwhile, ZmBAK1 may also activate downstream SA- and JA-related immune signaling pathways through interactions with different components, thereby positively regulating maize immunity to GSR.

In this study, we found that *F. graminearum* infection induced the expression of *ZmBAK1*. The genetic analysis using *bak1* mutant and overexpressor lines showed that ZmBAK1 is positively involved in the regulation of GSR resistance. It was also found that the activation of expression of multiple immune marker genes, as well as hall markers of PTI signaling pathway, including ROS burst and callose deposition were strongly activated in wild type, but deficient in the *zmbak1* mutant, suggesting that ZmBAK1-mediated GSR resistance is likely exerted via activating PTI signaling pathway in maize (Figure 5).

## Supplementary Material

Table S1.xlsx

Figure S1.tif

Figure S2.tif
