# Supplementary figures and images for: ZmBAK1 confers maize resistance to Gibberella stalk rot caused by Fusarium graminearum via activating PAMP-triggered immunity
Source: Plant Signal Behav. 2025 May 12;20(1):2502739. doi: 10.1080/15592324.2025.2502739 (PMC12077485; doi:10.1080/15592324.2025.2502739)

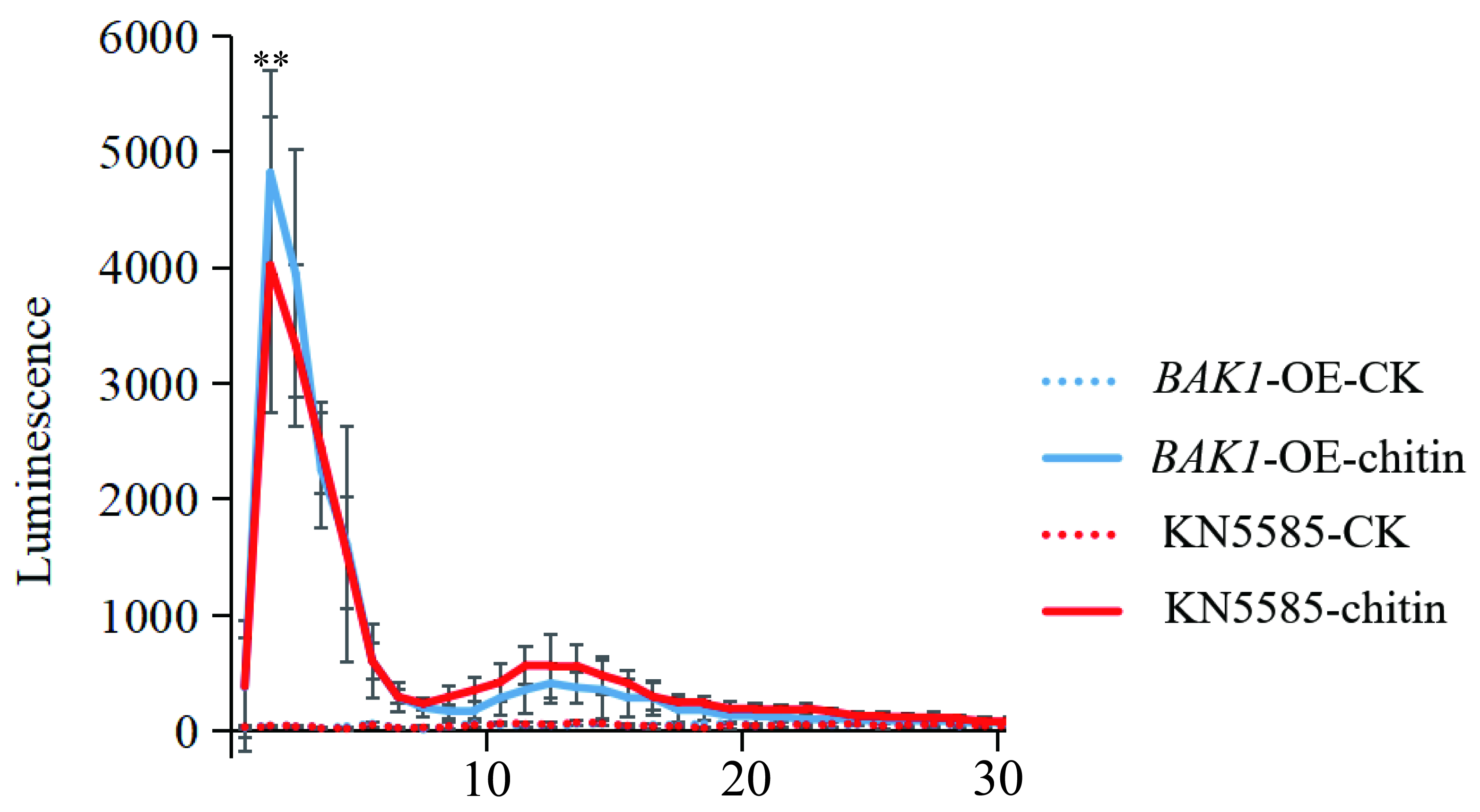

Supplement: Figure S1.tif [file KPSB_A_2502739_SM8381.tif]

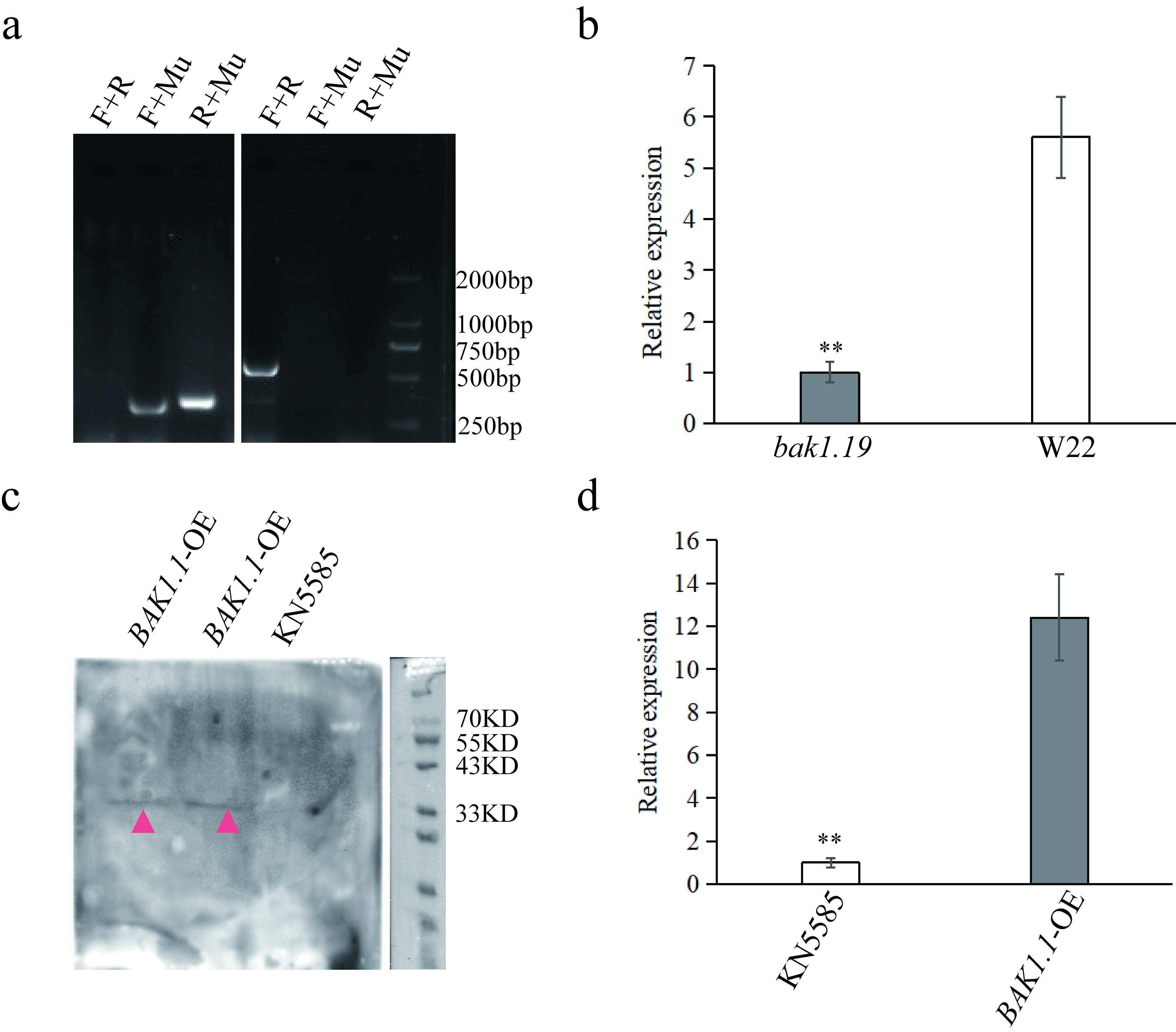

Supplement: Figure S2.tif [file KPSB_A_2502739_SM8380.tif]
